# Dietary Intervention Associated With Moderate-Intensity Continuous Training Leads to Changes in the Inflammatory Profile in Visceral Adipose Tissue but Not in Skeletal Muscle in Diet-Induced Obese Rats

**DOI:** 10.3389/fphys.2022.836484

**Published:** 2022-03-24

**Authors:** Jean Lucas Fernandes da Costa, Vanessa de Oliveira Furino, Cynthia Aparecida de Castro, João Manoel Alves, Ana Cláudia Garcia de Oliveira Duarte

**Affiliations:** ^1^Department of Physical Education and Human Motricity–DEFMH, Biological and Health Sciences Center–CCBS, Federal University of São Carlos–UFSCar, São Carlos, Brazil; ^2^Department of Morphology and Pathology-Biological and Health Sciences Center–CCBS, Federal University of São Carlos–UFSCar, São Carlos, Brazil; ^3^Department of Pharmacology, Ribeirão Preto Medical School, University of São Paulo, Ribeirão Preto, Brazil; ^4^Center of Research of Inflammatory Diseases, Ribeirão Preto Medical School, University of São Paulo, Ribeirão Preto, Brazil

**Keywords:** inflammatory markers, visceral adipose tissue, skeletal muscle, moderate-intensity continuous training, obesity

## Abstract

This study aimed to determine the concentrations of inflammatory markers in visceral adipose tissue (VAT) and skeletal muscle, and changes in body mass and adipocyte size in diet-induced obese rats after moderate-intensity continuous training (MICT) and/or dietary intervention. After 8 weeks of obesity induction through a high-fat diet (HFD) consumption, twenty diet-induced obese male Wistar rats were divided into four groups as follows: (i) control rats fed with HFD (HFD-SED), (ii) obese rats fed with HFD and submitted to MICT (HFD-MICT), (iii) obese rats that were submitted to a nutritional intervention by switching HFD to chow diet (CD-SED), and (iv) obese rats that were submitted to MICT and nutritional intervention (CD-MICT). All the animals in the training groups were submitted to MICT, with an intensity of 50–85% of V_*max*_, 60 min/day, 3 days/week for 8 weeks. Gastrocnemius muscle (GAST) and mesenteric adipose tissue (mWAT) were collected to quantify tumor necrosis factor alpha (TNF-α), interleukin (IL)-6, and IL-10 using ELISA. The body mass was recorded before and after the experimental protocols, and the adipocyte morphology was assessed using histological analysis. The results showed that HFD-SED had higher body mass, higher concentrations of inflammatory markers in mWAT, and higher increase in adipocyte size. The CD-SED and CD-MICT groups presented with reduced body mass, relative weight of mWAT, and adipocyte size. Moreover, the inflammatory markers in mWAT were reduced after dietary intervention (TNF-α), MICT (IL-10 and TNF-α), or both interventions combined (IL-6 and TNF-α). In contrast, there was no reduction in GAST-relative weight or concentrations of inflammatory markers for any treatment. Finally, we concluded that 8 weeks of dietary intervention alone and combined with MICT were effective in reducing some of the deleterious effects caused by obesity.

## Introduction

Changes in lifestyle, including a sedentary condition and consumption of an obesogenic diet (high fat and sugar), have contributed to the high prevalence of obesity and type 2 diabetes worldwide (Bray et al., 2016; Gopalan et al., 2021). The sedentary lifestyle associated with consuming a high-fat diet (HFD) induces a dynamic reprogramming in cellular metabolism, which affects insulin signaling and oxidative phosphorylation, and leads to metabolic syndrome (Chouchani and Kajimura, 2019). In addition, adipose tissue is a key driver of metabolic disturbance induced by obesity (Chouchani and Kajimura, 2019).

Excessive accumulation of visceral adipose tissue (VAT), which wraps around the internal organs (mesenteric fat), serves as the main site of inflammatory response through immune-cell infiltration and the release of inflammatory adipokines during positive energy balance conditions (Cao et al., 2021). Chronic low-grade inflammation in VAT involves an unhealthy expansion of adipocyte size (hypertrophy) (Jais and Brüning, 2017), local hypoxia, adipocyte death, mechanical stress, which leads to an excessive release of fatty acids and pro-inflammatory adipokines, such as interleukin (IL)-6 and tumor necrosis factor alpha (TNF-α), which are associated with metabolic disorders, including insulin resistance, hepatic steatosis, and cardiovascular diseases (Chouchani and Kajimura, 2019; Han et al., 2020; Kawai et al., 2021). Hence, adipose tissue serves as the hub of inflammation throughout the body (Zatterale et al., 2020). Thus, strategies that seek to recover from the inflammatory state of VAT are needed to restore cellular homeostasis and treat the obesity condition.

Aerobic exercise is an important non-pharmacological strategy to control obesity and can act as a preventative treatment against metabolic syndrome by stimulating visceral fat loss (Bellicha et al., 2021). The benefits of aerobic exercise on metabolic health include increased oxidative phosphorylation, improved insulin sensitivity, reduced release of pro-inflammatory adipokines, and the production of myokines that improve metabolic flexibility (Lancaster and Febbraio, 2014; Batatinha et al., 2019). In fact, it is well established that appropriate aerobic exercises stimulate the release of myokines with anti-inflammatory functions (Pedersen et al., 2007; Leal et al., 2018) and preserve the healthy function of adipose tissue (Lancaster and Febbraio, 2014). In addition, significant increases in the circulation of IL-6 during prolonged exercises were observed in previous studies (Steensberg et al., 2000, 2003), and further findings detected other myokines that are involved in this process, such as IL-8, IL-1 receptor antagonist (IL-1ra), IL-15, and irisin (Bay and Pedersen, 2020; Gonzalez-Gil and Elizondo-Montemayor, 2020).

One of the widely used physical exercise modalities aiming to reduce the deleterious effects of obesity is moderate-intensity continuous training (MICT), which is characterized by the intensities between 50–75% of maximum heart rate (HR_*max*_) (Wewege et al., 2017), 65–70% of maximum speed (V_*max*_) (Khalafi et al., 2020), and 65–70% of maximal oxygen consumption (VO_2*max*_) (Wang et al., 2017). MICT has been shown to be effective in modulating the weight gain of obese animals (Wang et al., 2017), promoting improvements in maximum exercise capacity (MEC) (Khalafi et al., 2020), reducing adipocyte expansion (Lacerda et al., 2019; Khalafi et al., 2020), and producing anti-inflammatory effects in adipose tissue (Lira et al., 2012) and skeletal muscle (Gopalan et al., 2021).

Another non-pharmacological strategy used to control obesity is dietary intervention (Longo and Panda, 2016). These approaches can occur through changes in the time of food supply (Aouichat et al., 2020), reducing the amount of food offered (Lowe et al., 2020), or through modulation in the nutrient composition. Despite this, many of these studies carried out interventions that combined the induction of obesity by HFD concomitantly with the beginning of MICT training and/or performed dietary interventions based on calorie restrictions. However, a few of them sought to investigate the effects of MICT associated with a dietary intervention without diets based on caloric restriction after an obesity induction period (Furino et al., 2021).

In view of this, some remaining questions were as follows: “Is it necessary to have five MICT sessions per week?” “If we associate training in fewer weekly sessions with a dietary intervention, could it lead to the same adaptations?” “Would the magnitude be the same?” Thus, the aim of this study was to investigate the effects of three MICT weekly sessions, dietary intervention, or both interventions combined, on the modulation of the inflammatory profile (IL-6, TNF-α, and IL-10) of skeletal muscle and VAT in rats with diet-induced obesity. Therefore, we hypothesized that aerobic exercise and dietary intervention may attenuate the inflammatory response induced by obesity in adipose tissue and that the combination of training plus dietary intervention will be able to induce a better reduction in the inflammatory response as part of a regulatory loop that increases the release of myokines by skeletal muscle.

## Materials and Methods

### Ethic and Experimental Groups

Twenty male *Rattus norvegicus albinus* (Wistar) rats with an initial weight of ≃300 g (45-day-old) were kept in polypropylene cages (five animals per cage) with food and water provided *ad libitum*. The environmental conditions were controlled with temperature and humidity maintained at 22–24°C and 50–60%, respectively, and a photoperiod of 12 h light/dark cycle (lights on at 6 p.m.). The Ethics Committee on Animal Use at the Federal University of São Carlos (São Paulo, Brazil) approved all experimental procedures under protocol no. 7631210617.

### Experimental Design

All animals were subjected to the same HFD for 8 weeks. After 8 weeks of diet-induced obesity, the rats were randomly divided into four groups: (i) control rats fed with HFD (HFD-SED), (ii) obese rats fed with HFD and submitted to MICT (HFD-MICT), (iii) obese rats that were submitted to nutritional intervention by switching HFD for chow diet (CD-SED), and (iv) obese rats that were submitted to MICT and nutritional intervention (CD-MICT). The sedentary groups (CD-SED and HFD-SED) were kept in their cages during the experimental period without any type of exercise, and the groups that underwent training (CD-MICT and HFD-MICT) performed 8 weeks of treadmill running.

### Diets

The experimental groups received the standard rat chow diet Agromix^®^ (Jaboticabal, SP, Brazil) in pellet form, which contained ∼23 g of protein, ∼39 g of carbohydrates, ∼4.8 g of total fat, and ∼5 g of fiber per 100 g of diet, or a palatable HFD that consisted of standard rat chow plus peanuts, milk chocolate, and sweet biscuits at a proportion of 3:2:2:1 that was mixed in pellet form (Estadella et al., 2004), containing ∼18 g of protein, ∼33 g of carbohydrate, ∼20 g of total fat, and ∼3 g of fiber per 100 g of diet (Costa et al., 2021).

### Body Mass and Food Intake Measurement

The body mass was measured once a week, and food intake was calculated by the difference in weight between the amount of food offered and the amount of food remaining every 2–3 days.

### Moderate-Intensity Continuous Training Protocol

#### Adaptation

Adaptation aimed to minimize the stress that can occur with chronic physical training without promoting physiological adaptations resulting from training (Kunstetter et al., 2018). Thus, before starting the training protocol, in the 8 week of obesity induction, the animals were familiarized with running on the treadmill adapted for rats for 5 consecutive days (10–15 min/day; 6–10 m/min) (Furino et al., 2021). Furthermore, to select the animals that would compose the training groups (MICT), an evaluation of physical performance was carried out (Rachetti et al., 2013). Following the evaluation model proposed by Rachetti et al. (2013), the animals selected for the MICT groups were those that had the highest arithmetic mean (on a scale of 1–5) during 5 days of adaptation.

#### Maximal Exercise Capacity

To determine the running speed used during the physical training protocol and evaluate the adaptations generated by treadmill training, the maximal exercise capacity (MEC) test was performed. The animals were placed on the treadmill and allowed to adapt over a 5-min period of 6 m/min without elevation, and the speed was gradually increased by 2 m/min every 2 min until they were unable to maintain the running pattern through increases in treadmill speed (Souza et al., 2018). The 100% of MEC is defined as the maximum speed (in m/min) and is used to determine the intensity of training sessions. Additionally, the MEC test was performed in the fourth week to adjust the training intensity and after 48 h in the last training session. Throughout the procedure, electric shocks were not used as a form of stimulation, but mechanical pressure was applied to the distal part of the tail.

#### Moderate-Intensity Continuous Training Protocol

The training protocol consisted of running sessions on a treadmill adapted for rats, containing six individual tracks separated by bays made of acrylic, always between 8 and 12 a.m. respecting the animals’ light-dark cycle. The training protocol had a frequency of three weekly sessions, for 8 weeks, 60 min per session at an intensity of 50–80% of the maximum speed (V_*max*_). Each training session was divided into three parts: 10 min for warmup (0–5 min: 50% of V_*max*_; 5–10 min: 60% of V_*max*_); 40 min for the main part (65–80% of V_*max*_); and 10 min for cool down (50% of V_*max*_) (Furino et al., 2021).

### Experiment and Tissue Collection

Animals were euthanized by decapitation 48 h after the last treadmill running session. The mesenteric adipose tissue (mWAT) and gastrocnemius muscle (GAST) were immediately removed, weighed, and stored at −80°C for further analysis. Additionally, a sample of mWAT was excised and fixed in 10% formalin to perform histological analysis.

### Histological Analysis

After being excised and fixed in 10% formalin, the mWAT samples were dehydrated in a grade alcohol series and the paraffin-embedded samples were cut (5 μm) using a microtome and mounted on glass slides that were then stained with H&E. The slides that were stained with H&E were digitized in a scanner (Pannoramic Digital Slide Scanners system, 3DHISTECH, Ltd., Hungary). In this study, we randomly digitized five images for each animal at 20× objective (Pannoramic Viewer), and then the Adiposoft (v. 1.15) plug-in of ImageJ Fiji (v 2.0.0) (National Institutes of Health, United States) was used to quantify the size (μm^2^) of the adipocytes (Galarraga et al., 2012; Parlee et al., 2014).

### Assessment of Mesenteric Adipose Tissue and Gastrocnemius Muscle Tissue Protein and Cytokine Concentrations

The samples of mWAT (∼200 mg) and GAST (∼100 mg) were homogenized in 600 and 500 μl, respectively, with an extraction buffer containing SDS 0.1% (p/v), Triton 1% (v/v), Tris–HCl pH 7.8, 50 mM, NaCl 150 mM, EDTA 15 mM, EGTA 5 mM, and protease inhibitors Complete Mini Roche^®^ 1× (Sigma-Aldrich, St. Louis, MO, United States). The mWAT samples were homogenized by rapid shaking with ceramic beads (2 × 30 s at 6 m/s) and GAST (3 × 30 s at 4 m/s) using a high-speed benchtop homogenizer FastPrep-24™ (MP Biomedicals, CA, United States). Then, the homogenate was centrifuged for 10 min at 10,000 × *g* for mWAT, whereas GAST was centrifuged for 15 min at 10,621 × *g*, both at a temperature of 4°C (Eppendorf^®^ Centrifuge 5430/5430R, Germany). The supernatant was transferred to a sterile microtube and brought to −80°C for further analysis.

The protein quantification of the supernatant was determined through the colorimetric assay of proteins based on bicinchoninic acid (BCA, AR0146-500) following the specifications of the BCA Protein Assay Kit (Boster Biological Technology, Pleasanton, CA, United States) and read in an automated plate reader (SpectraMax i3x machine, Molecular Devices, San Jose, CA, United States). According to the manufacturer’s instructions, IL-6, TNF-α, and IL-10 concentrations measured in lysate tissues were determined by ELISA at a 1:2 dilution. The method followed the specifications of the corresponding BD Biosciences Pharmingen^®^ (San Diego, CA, United States) kits (IL-6: Cat. No. 550319; IL-10: Cat. No. 555134; TNF-α: Cat. No. 558535). The concentrations of the samples were calculated from the concentration curve of the cytokine patterns and the final concentrations of protein in the tissues and were expressed in pg/mg.

### Statistical Analyses

All statistical analyses were performed using the GraphPad Prism software (version 8.0.2). The data are presented as the mean ± standard error (SEM). Data normality was verified with the Kolmogorov-Smirnov test. Comparisons among all groups were made using two-way ANOVA. The Tukey’s *post-hoc* analysis was used when the two-way ANOVA detected a statistical difference to assess multiple comparisons. The level of significance was *p* < 0.05.

## Results

### Dietary Intervention, but Not Aerobic Exercise, Reduced Weight Gain in Obese Rats

To explore the role of aerobic exercise and dietary intervention in weight gain, Wistar rats were fed an HFD for 8 weeks and subject to aerobic exercise, diet switch, or both interventions for an additional 8 weeks. The changes in body mass (g) are shown in Table 1. Regarding the baseline values (week 0) and after 8 weeks of obesity induction with HFD (week 8), the body mass did not differ between the groups (*p* > 0.05). After 8 weeks of dietary intervention and/or MICT (week 16), the body mass decreased in the CD-SED (*p* = 0.002) and CD-MICT groups (*p* = 0.003) compared to the HFD-SED group. The table also shows the variation in percentage (Δ%) between weeks 8 and 16. Similarly, the CD-SED and CD-MICT groups showed a significantly lower variation in percentage compared to the HFD-SED group (*p* = 0.02 and 0.002, respectively). These data suggest that the HFD was efficient in increasing the body mass of animals, and this process was reduced by dietary intervention independently and combined with MICT. Regarding food consumption, no differences were found in the daily consumption (g/day) between the groups (*p* > 0.05). Furthermore, the CD-SED group showed a lower caloric intake (kcal/day) compared to the HFD-SED group (*p* = 0.009) (Table 1).

**TABLE 1:** Changes in body mass (g) and food intake.

Groups	Body mass (g)				Daily consumption (g/day)	Caloric intake (kcal/day)
	Week 0	Week 8	Week 16	Δ % (W^16^–W^8^)		
HFD-SED	400.2 ± 12.3	559.6 ± 28.3	641.7 ± 33.9	14.6 ± 1.6	23.33 ± 0.7	108.9 ± 3.4
HFD-MICT	392.8 ± 7.0	551.4 ± 20.1	588.6 ± 17.3	6.9 ± 1.9	21.38 ± 0.5	99.8 ± 2.7
CD-SED	359.6 ± 23.6	465.0 ± 40.6	470.4 ± 32.7^a^	2.0 ± 3.2^a^	23.30 ± 1.0	89.9 ± 4.1^a^
CD-MICT	370.0 ± 21.1	488.0 ± 32.7	474.8 ± 34.9^a^	−2.6 ± 3.6^a^	25.00 ± 1.3	96.4 ± 5.0

*Data presented as mean ± SEM (p < 0.05). HFD-SED, high-fat diet sedentary (n = 5); HFD-MICT, high-fat diet plus MICT (n = 5); CD-SED, chow diet sedentary (n = 5); CD-MICT, chow diet plus MICT (n = 5).*

*^a^ vs. HFD-SED.*

Regarding the relative weights of depots (g/100 of body mass), in the mWAT depot, the CD-SED and CD-MICT groups showed lower values compared to the HFD-SED group (*p* = 0.003 and 0.009, respectively). For GAST, no statistically significant differences were found between the groups (*p* > 0.05). All the data are shown in Table 2.

**TABLE 2:** Relative weight of depots (g/100 BM).

Relative weight of depots (g/100 of body mass)		
Groups	mWAT	GASTROC
HFD-SED	1.99 ± 0.32	0.43 ± 0.03
HFD-MICT	1.10 ± 0.18	0.47 ± 0.01
CD-SED	0.61 ± 0.09^a^	0.50 ± 0.00
CD-MICT	0.44 ± 0.05^a^	0.52 ± 0.01

*Data presented as mean ± SEM (p < 0.05). mWAT, mesenteric adipose tissue; GAST, gastrocnemius muscle; HFD-SED, high-fat diet sedentary (n = 5); HFD-MICT, high-fat diet plus MICT (n = 5); CD-SED, chow diet sedentary (n = 5); CD-MICT, chow diet plus MICT (n = 5).*

*^a^ vs. HFD-SED.*

### Moderate-Intensity Continuous Training Associated With Dietary Intervention Promoted Adaptations in the Maximum Exercise Capacity Parameters in Greater Magnitudes

In the pre-intervention period, in the HFD-MICT group, the values of time (*p* = 0.001), distance (*p* = 0.002), and MEC (*p* < 0.0001) were higher compared to the CD-MICT group. For the variables time, distance, and MEC, both training groups showed an improvement in the parameters after 8 weeks of training (*p* < 0.0001). The delta percentage changes (Δ%) in MEC are also shown in the table. The CD-MICT group showed significantly greater improvements compared to the HFD-MICT group (*p* = 0.001). All the data are shown in Table 3.

**TABLE 3:** Variables of exercise.

		HFD-MICT	CD-MICT
Pre-exercise	MEC (m/min)	26.80 ± 0.49^a^	21.20 ± 0.49
	Time to exhaustion (min)	21.20 ± 0.49^a^	16.00 ± 1.09
	Distance covered (m)	568.80 ± 21.48^a^	340.80 ± 29.56
Post-exercise	MEC (m/min)	33.60 ± 0.74^c^	34.40 ± 0.74^c^
	Time to exhaustion (min)	29.60 ± 0.74^c^	30.40 ± 0.67^c^
	Distance covered (m)	996.80 ± 47.68^c^	1047.60 ± 45.08^c^
Δ of MEC (%)		25.36 ± 1.80	62.72 ± 5.99^b^

*Data presented as mean ± SEM (p < 0.05). HFD-MICT, high-fat diet plus MICT (n = 5); CD-MICT, chow diet plus MICT (n = 5).*

*^a^ vs. CD-MICT.*

*^b^ vs. HFD-MICT.*

*^c^ Pre-exercise vs. post-exercise in the same group.*

### Dietary Intervention, Independently and Combined With Moderate-Intensity Continuous Training, Reduced the Adipocyte Hypertrophy Caused by High-Fat Diet and Sedentarism

**FIGURE 1:**
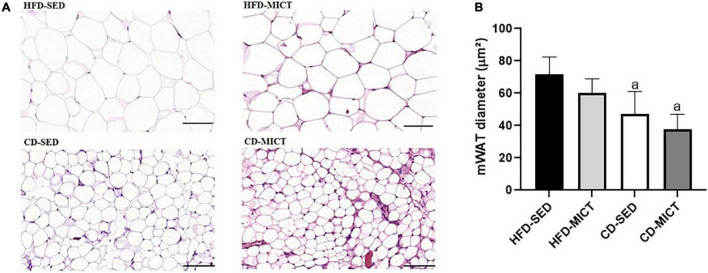
Photomicrograph of mesenteric adipose tissue (mWAT) after 8 weeks of dietary intervention and/or MICT. **(A)** Representative images of mWAT tissue showing H&E staining using 20× (100 μm) objective. **(B)** Diameter of mWAT adipocytes. HFD-SED, high-fat diet sedentary (*n* = 5); HFD-MICT, high-fat diet plus MICT (*n* = 4); CD-SED, chow diet sedentary (*n* = 5); CD-MICT, chow diet plus MICT (*n* = 4). Data presented as mean ± SEM (*p* < 0.05). *^a^* vs. HFD-SED.

The values of the adipocyte diameter (μm^2^) are shown in Figure 1. Similarly to body mass and the relative weight of mWAT depot, the CD-SED (47.16 ± 12.50 μm^2^, *p* = 0.01) and CD-MICT (37.59 ± 8.19 μm^2^, *p* = 0.002) groups showed a reduced adipocyte diameter compared to the HFD-SED group (71.72 ± 9.59 μm^2^), with no difference in the group that received only MICT (HFD-MICT, *p* = 0.58). These data suggest that the dietary intervention, independently or combined with MICT, is effective in reducing the size of adipocytes caused by consumption of the HFD and by sedentary lifestyle.

### Dietary Intervention, Independently and Associated With Moderate-Intensity Continuous Training, Was Efficient in Reducing the Concentration of Pro-inflammatory Markers in Mesenteric Adipose Tissue

**FIGURE 2:**
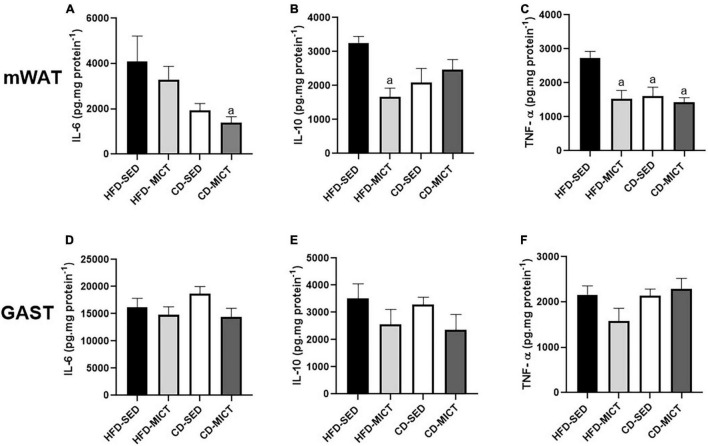
Mesenteric adipose tissue (mWAT) and gastrocnemius muscle (GAST) cytokines concentration. **(A,D)** IL-6, interleukin-6; **(B,E)** IL-10, interleukin-10; **(C,F)** TNF-α, tumor necrosis factor alpha. HFD-SED, high-fat diet sedentary (*n* = 5); HFD-MICT, high-fat diet plus MICT (*n* = 5); CD-SED, chow diet sedentary (*n* = 5); CD-MICT, chow diet plus MICT (*n* = 5). Data presented as mean ± SEM (*p* < 0.05). *^a^* vs. HFD-SED.

The cytokine concentrations (pg/mg) in mWAT and GAST are shown in Figure 2. For mWAT, the CD-MICT group showed lower IL-6 values compared to the HFD-SED group (*p* = 0.04) (Figure 2A). For IL-10 in mWAT, the group that received the intervention with training independently (HFD-MICT) showed reduced values of this cytokine compared to the HFD-SED group (*p* = 0.02) (Figure 2B). In addition, the values of TNF-α were reduced in the group that performed the training independently (HFD-MICT, *p* = 0.006), dietary intervention (CD-SED, *p* = 0.01), and the combination of both interventions (CD-MICT, *p* = 0.003) compared to the HFD-SED group (Figure 2C). The results demonstrate that the reduction in pro-inflammatory cytokines (IL-6 and TNF-α) in mWAT may have occurred as a result of a reduction in body adiposity, as well as a reduction in the expansion of adipocytes caused by dietary intervention and a combination of both treatments. For GAST (Figures 2D–F), no statistically significant differences were found (*p* > 0.05).

**FIGURE 3:**
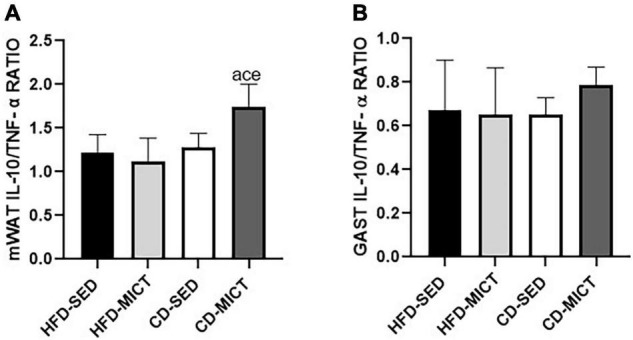
IL-10/TNF-α ratio. **(A)** Mesenteric adipose tissue (mWAT). **(B)** Gastrocnemius muscle (GAST). HFD-SED, high-fat diet sedentary (*n* = 5); HFD-MICT, high-fat diet plus MICT (*n* = 5); CD-SED, chow diet sedentary (*n* = 5); CD-MICT, chow diet plus MICT (*n* = 5). Data presented as mean ± SEM (*p* < 0.05). *^a^* vs. HFD-SED; *^c^* vs. HFD- MICT; *^e^* vs. CD-SED.

Figure 3 shows the values of IL-10/TNF-α ratio. For mWAT (Figure 3A), the CD-MICT group showed higher values compared to the HFD-SED (*p* = 0.01), HFD-MICT (*p* = 0.003), and CD-SED groups (*p* = 0.03), demonstrating a change in the proportion of pro- vs. anti-inflammatory cytokine concentration in this tissue, caused by the combination of both interventions. In contrast, for GAST (Figure 3B), no statistical difference was found between the groups (*p* > 0.05).

## Discussion

This study reports the effects of MICT, dietary intervention (associated or not) on body mass, adipocyte expansion, and the modulation of inflammatory markers in diet-induced obese rats. Our results showed the dietary intervention, independently and combined with MICT, was able to reduce the body mass, adipocyte expansion, and relative weight of mWAT, and also generated positive adaptations in the concentrations of inflammatory markers in this tissue, whereas for GAST no changes were observed.

The effects of MICT and dietary intervention, associated or independently, on body mass and other obesity parameters have been extensively investigated. Regarding MICT, the results in the literature are still controversial; while some studies did not show positive changes on body mass with MICT (Martinez-Huenchullan et al., 2019; Khalafi et al., 2020), some of them have shown that this training model significantly reduced the body mass of obese animals (Wang et al., 2017). In the same way, MICT has independently been shown to be an important strategy to reduce adipocyte expansion (Lacerda et al., 2019; Khalafi et al., 2020).

In contrast to these previous studies, our findings indicated that MICT alone did not promote decreases in body mass, mWAT relative weight, and adipocyte hypertrophy of obese animals. Although a 30% reduction in the diameter of adipocytes of the HFD-MICT animals compared to the HFD-SED group was observed, this difference was not statistically significant (*p* = 0.44). These data can be associated with a reduced weekly training frequency in our protocol (three weekly sessions) compared to the protocols previously applied in the literature (five weekly sessions, 65–70 of VO_2*max*_) (Wang et al., 2017; Khalafi et al., 2020), suggesting that our protocol may not have increased in resting energy expenditure and resulted in a negative energy balance enough to generate such adaptations in adiposity (Lacerda et al., 2019; Khalafi et al., 2020). In addition, these results may be related to the fact that the mesenteric fat increases the lipogenesis *de novo* as a means to replace the lipids that are utilized during the exercise, hindering the loss of body mass and reduction in the area of adipocytes (Lehnig et al., 2019).

Furthermore, studies have shown that caloric restriction reduces body adiposity parameters due to the negative energy balance (Miller et al., 2017; Vangoitsenhoven et al., 2018). Moreover, the association between training and dietary interventions has a greater impact on body mass compared to the dietary intervention and exercise alone (Verheggen et al., 2016). Our results show that the dietary intervention used in this protocol (Δ14.2%) was effective in reducing the body mass, mWAT relative weight, and adipocyte size, with less marked changes in the feeding patterns. These results are reinforced by the literature, which showed that in diet-induced obese rats the consumption of 9% fat diet, or the return to a chow diet, was able to reduce the adipocyte size of obese rats (Vangoitsenhoven et al., 2018; Hansson et al., 2019).

Although no change in obesity parameters was observed in the HFD-MICT group, we also investigated other adaptations related to the treatments. In our study, both the MICT groups showed an increase in MEC compared to the pre-training results. However, the HFD-MICT group presented improvements in a smaller magnitude compared to the CD-MICT group. These results were previously attributed to the HFD effects on the metabolism of animals such as (a) a rapid accumulation of blood lactate due to a downregulation in lactate dehydrogenase isoform B (LDHB) and a decrease in monocarboxylate transporters 2 (MCT2) (Chen et al., 2017); (b) an alteration in glucose metabolism, which induces insulin resistance and glucose intolerance (Mardare et al., 2016); and (c) impairment of mitochondrial function in skeletal muscle (Takada et al., 2015). In our study, it is possible that these physiological aspects may have influenced the performance of the HFD-MICT group at the end of the experimental period, mainly on the deleterious effects of HFD on skeletal muscle (Ribeiro et al., 2019).

To consolidate the obese metabolic profile, we also evaluated the concentration of inflammatory cytokines. We observed that at 16 weeks of palatable HFD, increases in the concentration of inflammatory markers (IL-6, IL-10, and TNF-α) in the mWAT of the HFD-SED group were observed. These values were reduced after 8 weeks of dietary intervention (TNF-α), MICT (IL-10 and TNF-α), or both interventions (IL-6 and TNF-α). This behavior occurs, along with the reduction of body weight, adipocyte hypertrophy, and decrease in the relative weight of mWAT, and may be associated with an increase in lipolysis and a decrease in lipogenesis related to the irregular effect of these cytokines (e.g., IL-6 and TNF-α) during obesogenic processes (Jeong et al., 2015; Gonzalez-Gil and Elizondo-Montemayor, 2020).

Regarding pro-inflammatory cytokines, IL-10 presents an anti-inflammatory characteristic and plays an important role in the modulation of inflammatory processes during obesity, mainly through the regulation and reduction of the inflammatory process caused by physical exercises (Lira et al., 2009a,b; Rocha-Rodrigues et al., 2017). Our findings showed that the concentration of IL-10 was reduced in the HFD-MICT group compared to the HFD-SED group. This higher concentration in the HFD-SED group may have been a consequence of the increase in the production of pro-inflammatory cytokines (e.g., TNF-α and IL-6) in order to minimize the inflammatory status (Esposito et al., 2003; Juge-Aubry et al., 2005; Yamashita et al., 2010). Furthermore, a decrease in the concentrations of this cytokine caused by exercises may have occurred for the same reason. These counterregulation effects occur because IL-10 can stimulate its own production, inhibiting the production of inflammatory cytokines (e.g., IL-1β, TNF-α, and IL-6) and also increasing the release of soluble TNF receptors, which antagonize the effects of TNF-α on adipose tissue (Yamashita et al., 2010).

Furthermore, the IL-10/TNF-α ratio was used in several studies as an indicator of the inflammatory status (Kaur et al., 2006; Leonidou et al., 2007; Jung et al., 2008; Lira et al., 2012). Previous studies have shown that aerobic training was able to promote increases in this ratio, both in serum (Chagas et al., 2017; Rocha-Rodrigues et al., 2017) and/or tissue levels (Lira et al., 2009a,^2012^). On the other hand, our findings demonstrated that this effect resulted only due to the association of dietary intervention and MICT, suggesting that both interventions combined present an anti-inflammatory effect on mWAT. While in the GAST, since we did not see changes in cytokine concentrations, it was considered that the ratio would not change.

Furthermore, another mechanism that could explain the benefits of aerobic training in obese animals is the release of myokines. Some studies have reported the anti-inflammatory effects of physical exercise mediated by myokines, regardless of decreases in fat (Rosa-Neto et al., 2009; Balducci et al., 2012; Gopalan et al., 2021). In addition, exercising modalities such as running (Gopalan et al., 2021) or swimming (Shirvani et al., 2021) were shown to be important in reducing the inflammatory state of the muscle tissue due to the decrease in the deposition of ectopic fat in this compartment caused by training or by the downregulation of the expression of TLR4/MyD88. The protocols and/or time of interventions in our study may have been insufficient to reduce fat deposition in this compartment because no differences were found in the relative muscle mass between the groups. However, due to the particularities of our protocols and the absence of a histological analysis to verify the deposition of fat in skeletal muscle, it is impossible to clearly affirm which mechanisms were involved in these processes.

This study has some limitations as follows: (1) the number of samples per group was limited to five animals. It is possible that a larger sample size would elicit stronger conclusions. (2) We did not perform histological analyses of GAST to check the deposition of ectopic fat in this tissue or serum concentrations of inflammatory or anti-inflammatory markers to add these parameters during our investigation. Hence, new perspectives for future studies with a larger number of animals per group, with other analysis and a longer time of intervention to investigate additional cytokines/myokines, are suggested. In conclusion, we found that the reduction in adiposity, the concentration of inflammatory markers in mWAT, and increases in MEC in greater magnitudes play a major role in the benefits induced by dietary intervention, combined or not with MICT, demonstrating that dietary intervention and the combination of both treatments are effective in reducing some of the deleterious effects caused by obesity.

## Data Availability Statement

The original contributions presented in the study are included in the article/supplementary material, further inquiries can be directed to the corresponding author.

## Ethics Statement

The animal study was reviewed and approved by the Ethics Committee on the Use of Animals (CEUA) of Federal University of São Carlos (São Paulo, Brazil) approved all experimental procedures under protocol no. 7631210617.

## Author Contributions

JC, VF, and AD helped conceive the design, performed the analyses, analyzed the data, and wrote the first draft of the manuscript and helped conceive the design, helped with the data analyses, provided funding for the study, and helped draft the manuscript. JC, VF, and JA helped to conceive the design and supervised the experimental trials and training sessions. JC, VF, and CC helped with other data analyses and helped draft the manuscript. JC, VF, CC, JA, and AD interpreted the study results and edited the manuscript. All authors have read and approved the final version of the manuscript and agreed with the order of presentation of the authors.

## Conflict of Interest

The authors declare that the research was conducted in the absence of any commercial or financial relationships that could be construed as a potential conflict of interest.

## Publisher’s Note

All claims expressed in this article are solely those of the authors and do not necessarily represent those of their affiliated organizations, or those of the publisher, the editors and the reviewers. Any product that may be evaluated in this article, or claim that may be made by its manufacturer, is not guaranteed or endorsed by the publisher.
